# Buckling Behavior of Nanobeams Placed in Electromagnetic Field Using Shifted Chebyshev Polynomials-Based Rayleigh-Ritz Method

**DOI:** 10.3390/nano9091326

**Published:** 2019-09-16

**Authors:** Subrat Kumar Jena, Snehashish Chakraverty, Francesco Tornabene

**Affiliations:** 1 Department of Mathematics, National Institute of Technology Rourkela, Rourkela 769008, India; sjena430@gmail.com (S.K.J.); sne_chak@yahoo.com (S.C.); 2 Department of Innovation Engineering, University of Salento, 73100 Lecce, Italy

**Keywords:** buckling, electromagnetic field, nanobeam, shifted chebyshev polynomial, rayleigh-ritz method

## Abstract

In the present investigation, the buckling behavior of Euler–Bernoulli nanobeam, which is placed in an electro-magnetic field, is investigated in the framework of Eringen’s nonlocal theory. Critical buckling load for all the classical boundary conditions such as “Pined–Pined (P-P), Clamped–Pined (C-P), Clamped–Clamped (C-C), and Clamped-Free (C-F)” are obtained using shifted Chebyshev polynomials-based Rayleigh-Ritz method. The main advantage of the shifted Chebyshev polynomials is that it does not make the system ill-conditioning with the higher number of terms in the approximation due to the orthogonality of the functions. Validation and convergence studies of the model have been carried out for different cases. Also, a closed-form solution has been obtained for the “Pined–Pined (P-P)” boundary condition using Navier’s technique, and the numerical results obtained for the “Pined–Pined (P-P)” boundary condition are validated with a closed-form solution. Further, the effects of various scaling parameters on the critical buckling load have been explored, and new results are presented as Figures and Tables. Finally, buckling mode shapes are also plotted to show the sensitiveness of the critical buckling load.

## 1. Introduction

In recent decades, with the advent of technological advancement, small-scale structures like nanoclock, nano-oscillator, nanosensor, NEMS (Nano-Electro-Mechanica System), and so forth have found tremendous attention due to their various applications. In this scenario, the study of dynamical behaviors of nanostructures is important and a need of the hour. Due to the small size of nanostructures, experimental analysis of such structures is always challenging and difficult as it requires enormous experimental efforts. Moreover, classical mechanics or continuum mechanics fail to address the nonlocal effect also. In this regard, nonlocal continuum theories were recently found to be useful for the modeling of micro- and nanosized structures. Various researchers developed different nonlocal or nonclassical continuum theories to assimilate the small-scale effect, such as strain gradient theory [1], couple stress theory [2], micropolar theory [3], nonlocal elasticity theory [4], and so on. Out of all these theories, nonlocal elasticity theory of Eringen has been broadly used for the dynamic analysis of nanostructures. Few studies regarding the vibration and buckling of beam, membrane, and nanostructures such as nanobeam, nanotube, nanoribbon, and so forth can be found in [5,6,7,8,9,10,11,12,13,14].

Wang et al. [15] studied buckling behavior of micro- and nanorods/tubes with the help of Timoshenko beam theory, where small-scale effect was addressed by using the nonlocal elasticity theory of Eringen. Emam [16] incorporated nonlocal elasticity theory to analyze the buckling and the postbuckling response of nanobeams analytically. Yu et al. [17] used nonlocal thermo-elasticity theory to study buckling behavior of Euler–Bernoulli nanobeam with nonuniform temperature distribution. Nejad et al. [18] employed a generalized differential quadrature method to undertake the buckling analysis of the nanobeams made of two-directional functionally graded materials (FGM) using nonlocal elasticity theory. Dai et al. [19] analytically studied the prebuckling and postbuckling behavior of nonlocal nanobeams subjected to the axial and longitudinal magnetic forces. Bakhshi Khaniki and Hosseini-Hashemi [20] implemented nonlocal strain gradient theory to investigate the buckling behavior of Euler–Bernoulli beam, considering different types of cross-section variation using the generalized differential quadrature method. Yu et al. [21] proposed a three characteristic-lengths-featured size-dependent gradient-beam model by incorporating the modified nonlocal theory and Euler–Bernoulli beam theory. He implemented the weighted residual approach to solve the six-order differential equation to investigate the buckling behaviors. Malikan [22] used a refined beam theory to investigate the buckling behavior of SWCNT (Single Walled carbon NanoTube) using Navier’s method. Here, unidirectional load is applied on the SWCNT. Buckling analysis of FG (Fanctionally Graded) nanobeam was studied in [23] analytically with the help of Navier’s method under the framework of first-order shear deformation beam theory. Malikan et al. investigated the transient response [24] of nanotube for a simply supported boundary condition using Kelvin–Voigt viscoelasticity model with nonlocal strain gradient theory. An investigation regarding damped forced vibration of SWCNTs using a shear deformation beam theory subjected to viscoelastic foundation and thermal environment can be found in [25]. Some other notable studies can be seen in [26,27,28,29,30] 

As per the present authors’ knowledge, the buckling behavior of the Euler–Bernoulli nanobeam placed in an electro-magnetic field using shifted Chebyshev polynomials Rayleigh-Ritz method has been studied for the first time for “Pined–Pined (P-P), Clamped–Pined (C-P), Clamped–Clamped (C-C), and Clamped-Free (C-F)” boundary conditions. Euler–Bernoulli nanobeam is combined with Hamilton’s principle to derive the governing equation. Also, a closed-form solution for the Pined–Pined (P-P) boundary condition has been obtained by using the Navier’s technique. Critical buckling load for all the classical boundary conditions were obtained and a parametric study has been carried out to comprehend the effects of various scaling parameters on the critical buckling load through graphical and tabular results. Further, buckling mode shapes for different boundary conditions were drawn to show the sensitivity towards various scaling parameters.

## 2. Proposed Model for Electromagnetic Nanobeam

**Figure 1:**
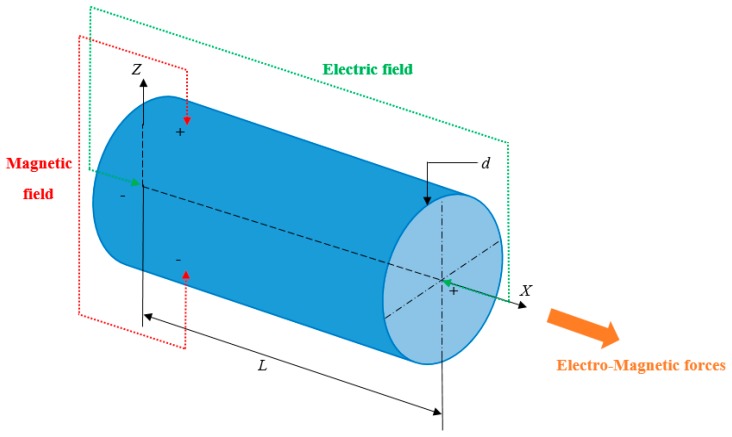
Schematic continuum model of the nanobeam placed in electromagnetic field.

In this study, the nanobeam with length L and diameter d is placed in an electromagnetic field with the electric field intensity as Ε and the magnetic flux density as Β. The schematic diagram for continuum model of the nanobeam is shown in Figure 1. Then, by Ohm’s law, the current density (J) of the system due to the induced current (because of Lorentz force) is given as [31]

(1) $$J=\sigma_{0}\left(Ε+w_{0}\times Β\right)=\sigma_{0}\left(Ε+w_{0}\times \mu_{0}H\right)$$

where $\sigma_{0}$ is the electrical conductivity, $\mu_{0}$ is the magnetic permeability of free space, and H is the magnetic field strength. By neglecting the electric field intensity, the nanobeam experiences a magnetic force or Pondermotive force which is denoted by $f_{em}$ and can be expressed as [31]

(2) $$f_{em}=J\times Β=\sigma_{0}\left(Ε+w_{0}\times \mu_{0}H\right)\times \mu_{0}H=\sigma_{0}\mu_{0}^{2}H^{2}w_{0}$$

According to Euler–Bernoulli beam theory, the displacement field at any point may be stated as [32]

(3a) $$u_{1}\left(x,\;z,\;t\right)=\;-z\frac{\partial w_{0}\;\left(x,\;t\right)}{\partial x}$$

(3b) $$u_{3}\left(x,\;z,\;t\right)=w_{0}\;\left(x,\;t\right)$$

Here, $u_{1}$ and $u_{3}$ represent displacements along x and z directions, respectively, and $w_{0}\;\left(x,\;t\right)$ denotes the transverse displacement of the point on the mid-plane of the beam. The strain-displacement relation may be expressed as

(4) $$\varepsilon_{xx}=-z\frac{\partial^{2}w_{0}\;\left(x,\;t\right)}{\partial x^{2}}$$

Under the framework of Euler–Bernoulli nanobeam, the variation of strain energy (δU) and the variation of work done by external force $\left(\delta W_{e}\right)$ are presented as

(5) $$\delta U=\int_{0}^{L}\int_{A}\sigma_{xx}\delta \varepsilon_{xx}dA\;dx=\int_{0}^{L}\left[-M_{xx}\frac{\partial^{2}\delta w_{0}}{\partial x^{2}}\right]\;dx,$$

(6) $$\delta W_{e}=\int_{0}^{L}\left[P\;\left(\frac{dw_{0}}{dx}\right)\left(\frac{d\delta w_{0}}{dx}\right)+\sigma_{0}\mu_{0}^{2}H^{2}w_{0}\delta w_{0}\right]\;dx,$$

where $\sigma_{xx}$ is the normal stress, $\varepsilon_{xx}$ is the normal strain, and $M_{xx}=\int_{A}\sigma_{xx}\;z\;dA$ is the bending moment of nanobeam. The Hamilton’s principle for the conservative system is stated as

(7) $$\delta \prod =\int_{0}^{t}\delta \;\left(W_{e}+U\right)\;dt,$$

Substituting Equations (5)–(7) and setting δ∏=0, we have

(8) $$\begin{array}{ll} \delta \prod & =\int_{0}^{t}\int_{0}^{L}\left[P\;\left(\frac{dw_{0}}{dx}\right)\left(\frac{d\delta w_{0}}{dx}\right)+\sigma_{0}\mu_{0}^{2}H^{2}w_{0}\delta w_{0}-M_{xx}\frac{\partial^{2}\delta w_{0}}{\partial x^{2}}\right]\;dxdt\\ & =\int_{0}^{t}\int_{0}^{L}\left[-P\;\left(\frac{d^{2}w_{0}}{dx^{2}}\right)\delta w_{0}+\sigma_{0}\mu_{0}^{2}H^{2}w_{0}\delta w_{0}+\frac{\partial^{2}M_{xx}}{\partial x^{2}}\delta w_{0}\right]\;dxdt \end{array}$$

The equation of motion for buckling behavior can be obtained as 

(9) $$\frac{d^{2}M_{xx}}{dx^{2}}+\sigma_{0}\mu_{0}^{2}H^{2}w_{0}=P\;\frac{d^{2}w_{0}}{dx^{2}}$$

For an isotropic nonlocal beam, the nonlocal elasticity theory of Eringen can be expressed as [4]

(10) $$\left(1-\mu \frac{\partial^{2}}{\partial x^{2}}\right)\sigma_{xx}=E\varepsilon_{xx}$$

where $\mu ={\left(e_{0}a\right)}^{2}$ is the nonlocal parameter, E is Young’s modulus. Here $e_{0}$ and a denote material constant and internal characteristic length, respectively. Multiplying Equation (10) by zdA and integrating over A, the nonlocal constitutive relation for Euler–Bernoulli nanobeam may be expressed as

(11) $$M_{xx}-\mu \frac{d^{2}M_{xx}}{dx^{2}}=-EI\frac{d^{2}w_{0}}{dx^{2}}$$

where $I=\int_{A}z^{2}dA$, is the second moment of area. Using Equation (9) in Equation (11) and rearranging, the nonlocal bending moment can be obtained as

(12) $$M_{xx}=-EI\frac{d^{2}w_{0}}{dx^{2}}+\mu P\frac{d^{2}w_{0}}{dx^{2}}-\mu \sigma_{0}\mu_{0}^{2}H^{2}w_{0}$$

Equating the nonlocal strain energy with work done by an external force, we obtain

(13) $$-\int_{0}^{L}\left(-EI\frac{d^{2}w_{0}}{dx^{2}}+\mu P\frac{d^{2}w_{0}}{dx^{2}}-\mu \sigma_{0}\mu_{0}^{2}H^{2}w_{0}\right)\frac{d^{2}w_{0}}{dx^{2}}\;dx=\int_{0}^{L}\left[P\;{\left(\frac{dw_{0}}{dx}\right)}^{2}+\sigma_{0}\mu_{0}^{2}H^{2}w_{0}^{2}\right]\;dx$$

Substituting Equation (12) in Equation (9), we obtain the governing equation of motion as

(14) $$-EI\frac{d^{4}w_{0}}{dx^{4}}+\mu P\frac{d^{4}w_{0}}{dx^{4}}-\mu \sigma_{0}\mu_{0}^{2}H^{2}\frac{d^{2}w_{0}}{dx^{2}}+\sigma_{0}\mu_{0}^{2}H^{2}w_{0}=p\frac{d^{2}w_{0}}{dx^{2}}$$

Let us define the following nondimensional parameters 

$X=\frac{x}{L}\;$ = nondimensional spatial coordinate

$W=\frac{w_{0}}{L}=$ nondimensional transverse displacement

$\hat{P}=\frac{PL^{2}}{EI}=$ dimensionless frequency parameter

$\alpha =\frac{e_{0}\alpha }{L}=$ dimensionless nonlocal parameter

$H_{a}^{2}=\frac{\sigma_{0}\mu_{0}^{2}H^{2}L^{4}}{EI}=$ dimensionless Hartmann parameter.

Incorporating the above nondimensional parameters in Equations (13) and (14), we have

(15) $$\int_{0}^{1}\left\{{\left(\frac{d^{2}W}{dX^{2}}\right)}^{2}+\alpha^{2}H_{a}^{2}\left(W\frac{d^{2}W}{dX^{2}}\right)-H_{a}^{2}W^{2}\right\}\;dX=\hat{P}\int_{0}^{1}\left\{{\left(\frac{dW}{dX}\right)}^{2}+\alpha^{2}{\left(\frac{d^{2}W}{dX^{2}}\right)}^{2}\right\}dX$$

(16) $$\frac{d^{4}W}{dX^{4}}+\alpha^{2}H_{a}^{2}\frac{d^{2}W}{dX^{2}}-H_{a}^{2}W=\hat{P}\;\left(\alpha^{2}\frac{d^{4}W}{dX^{4}}-\frac{d^{2}W}{dX^{2}}\right)$$

## 3. Solution Methodology

### 3.1. Shifted Chebyshev Polynomials-Based Rayleigh-Ritz Method

Chebyshev polynomials of the first kind $\left(C_{n}\left(X\right)\right)$ are a sequence of orthogonal polynomials with X∈[−1 1], and few terms of the sequence are defined as

$$C_{0}(X)=1$$

(17) $$C_{1}(X)=X$$

$$C_{n}(X)=2XC_{n-1}(X)-C_{n-2}(X),\;n=2,\;3,\;\ldots$$

In order to solve Equation (12), Rayleigh-Ritz method is implemented along with Chebyshev polynomials of the first kind as shape function. For more details about the Rayleigh-Ritz method, one may refer to the books [33,34]. The main advantages of using Chebyshev polynomials over algebraic polynomials $\left(1,\;X,\;X^{2},\;X^{3},\;\ldots X^{n}\right)$ are the orthogonal properties of Chebyshev polynomials, which reduce the computational effort, and for the higher value of n (n>10), the system avoids ill-conditioning. Since the domain of the nanobeam lies in [0 1], the Chebyshev polynomials must be reduced to shifted Chebyshev polynomials of the first kind $\left(C_{n}^{*}\left(X\right)\right)$ with X∈[0 1]. This is achieved by transforming X↦2X−1, and there exists a one-to-one correspondence between [0 1] and [−1 1]. Accordingly, the first few terms of shifted Chebyshev polynomials of the first kind $\left(C_{n}^{*}\left(X\right)\right)$ can be written as, (where $C_{n}^{*}\left(X\right)=C_{n}(2X-1)$)

$$C_{0}^{*}(X)=1$$

(18) $$C_{1}^{*}(X)=2X-1$$

$$C_{n}^{*}(X)=2(2X-1)C_{n-1}^{*}(X)-C_{n-2}^{*}(X),\;n=2,\;3,\;\ldots$$

The transverse displacement function (W(X)) as per the Rayleigh-Ritz method can now be expressed as

(19) $$W\left(X\right)=X^{p}{\left(1-X\right)}^{q}\sum_{i=1}^{N}a_{i}C_{i-1}^{*}(X)$$

where $a_{i}\prime s$ are unknowns, $C_{i-1}^{*}$ are the shifted Chebyshev polynomials of the index i−1, N is the number of terms required to obtain the result with the anticipated accuracy, p and q are the exponents which decide the boundary conditions, as given in Table 1.

Replacing Equation (19) into Equation (15), and minimizing the buckling load parameter with respect to the coefficients of the admissible functions (i.e., $a_{i}\prime s,\;i=1,\;2,\;3\;\ldots N\;$), we obtain the generalized eigenvalue problem as

(20) $$\left[Κ\right]\;\left\{A\right\}=\hat{P}\;\left[Β\right]\;\left\{A\right\}$$

where $A={\left[a_{1}\;a_{2}\;a_{3}\;\ldots a_{N}\right]}^{T}$, [Κ] is the stiffness matrix and [Β] is the buckling matrix, which are presented as

$$Κ\left(i,\;j\right)=\int_{0}^{1}\left(2C_{i}^{*}{}^{\prime \prime }C_{j}^{*}{}^{\prime \prime }+\alpha^{2}H_{a}^{2}C_{i}^{*}{}^{\prime \prime }C_{j}^{*}+\alpha^{2}H_{a}^{2}C_{i}^{*}C_{j}^{*}{}^{\prime \prime }-2H_{a}^{2}C_{i}^{*}C_{j}^{*}\right)\;dX,\;i,\;j=1,\;2,\;3,\ldots N$$

$$Β\left(i,\;j\right)=\int_{0}^{1}\left(2C_{i}^{*}{}^{\prime }C_{j}^{*}{}^{\prime }+2\alpha^{2}C_{i}^{*}{}^{\prime \prime }C_{j}^{*}{}^{\prime \prime }\right)\;dX,\;i,\;j=1,\;2,\;3,\ldots N.$$

### 3.2. Closed-Form Solution for P-P Boundary Condition Using Navier’s Technique

Navier’s technique has been employed to find a closed-form solution for the Pined–Pined (P-P) boundary condition. As per the Navier’s technique, the transverse displacement (W) may be expressed as [23,24,25]

(21) $$W\;=\sum_{n=1}^{\infty }W_{n}\;\sin\left(n\pi X\right)\;e^{i\omega_{n}t}$$

In which $W_{n},\;$ and $\omega_{n}$ are the displacement and frequency of the beam. Now, by substituting Equation (21) in Equation (16), the buckling load $\hat{P}$ for Pined–Pined (P-P) boundary condition is calculated as

(22) $${\hat{P}}_{n}=\frac{{\left(n\pi \right)}^{4}-\alpha^{2}H_{a}^{2}{\left(n\pi \right)}^{2}-H_{a}^{2}}{\alpha^{2}{\left(n\pi \right)}^{4}+{\left(n\pi \right)}^{2}}$$

## 4. Numerical Results and Discussion

Shifted Chebyshev polynomials-based Rayleigh-Ritz method has been employed to convert Equation (15) into the generalized eigenvalue problem given in Equation (20). MATLAB codes have been utilized to solve the generalized eigenvalue problem and to compute the critical buckling load parameter. Likewise, Navier’s technique has been adopted to find a closed-form solution for the P-P boundary condition, which is demonstrated in Equation (22). In this regard, the following parameters are taken from [15] for computation purpose

E=1 Tpa, d=1 nm, and L=10 nm.

### 4.1. Validation

Results of the present model were authenticated by two ways, firstly, by matching with the numerical results given by Wang et al. (2006) for “P-P, C-P, C-C, and C-F” boundary conditions and secondly, Pined–Pined results were compared with the closed-form solution obtained by Navier’s technique. For this purpose, the Hartmann parameter $\left(H_{a}\right)$ in the present model was taken as zero, and the critical buckling load parameters $\left(P_{cr}\right)$ for “P-P, C-P, C-C, and C-F” boundary conditions were taken into investigation. Comparisons of critical buckling load $\left(P_{cr}\right)$ are presented in Table 2. Similarly, Table 3 illustrates the comparison of the P-P boundary condition with the Navier’s results, with E=1 TPa, d=1 nm, L=10 nm, and $H_{a}=1$. From these comparisons, it is evident that the critical buckling loads of the present model are on a par with [15] in the particular case and with Navier’s solution for the P-P boundary condition.

### 4.2. Convergence

**Figure 2:**
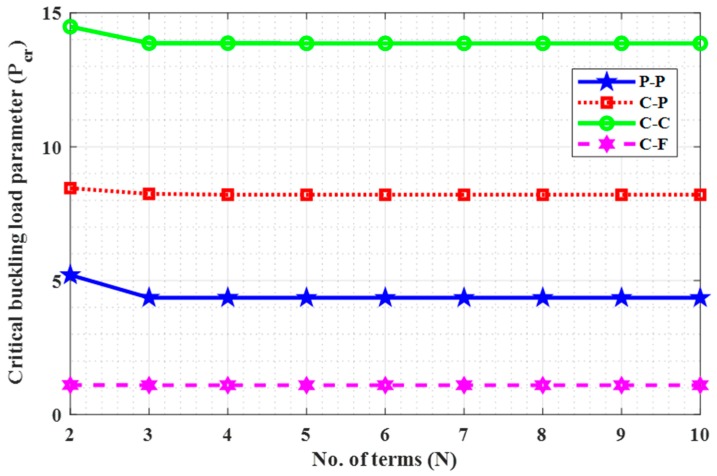
No. of terms (N) vs. critical buckling load $\left(P_{cr}\right)$ with $H_{a}=1$, L=10, and $e_{0}a=1$.

A convergence study has been performed to know the number of terms needed to obtain the results of critical buckling load parameters $\left(P_{cr}\right)$ and verify the present model using the Rayleigh-Ritz method. In this regard, $H_{a}=1$, L=10, and $e_{0}a=1$ were taken for computation purpose. Both the tabular and graphical results were noted for “P-P, C-P, C-C, and C-F” boundary conditions, which are demonstrated in Table 4 and Figure 2, respectively. The C-F boundary condition is converging faster with N=5, whereas other edges such as P-P, C-P, and C-C take N=7 for acquiring the desired accuracy. These results revealed that both the model and the results are useful regarding the present investigation.

### 4.3. Influence of Small Scale Parameter

**Figure 3:**
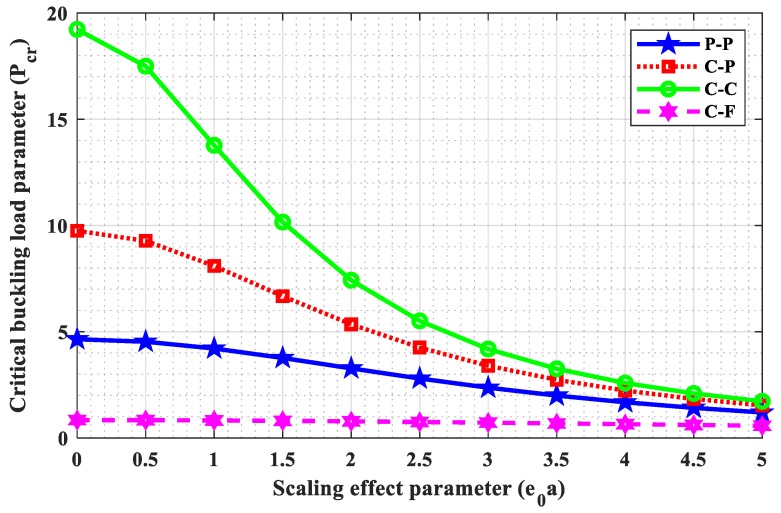
Variation of $\left(e_{0}a\right)$ with $\left(P_{cr}\right)$ for N=7, L=10, and $H_{a}=2$.

**Figure 4:**
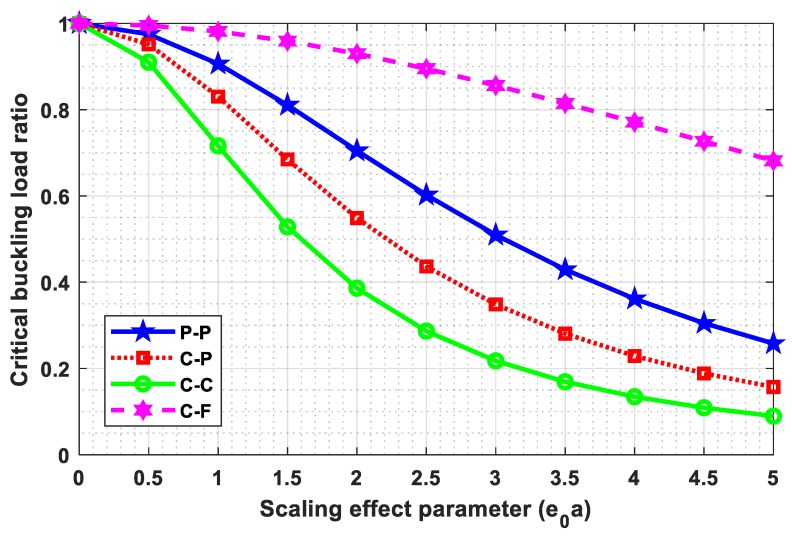
Small scale parameter $\left(e_{0}a\right)$ vs. critical buckling load ratio.

This subsection is dedicated to investigating the influence of a small scale parameter $\left(e_{0}a\right)$ on critical buckling load parameters and the critical buckling load ratio. The four frequently used boundary conditions such as “P-P, C-P, C-C, and C-F” were taken into consideration with N=7, L=10, and $H_{a}=2$. Tabular and graphical results are illustrated in Table 4 and Figure 2 and Figure 3 for different $e_{0}a$ (0, 0.5, 1, 1.5, 2, 2.5, 3, 3.5, 4, 4.5, 5). Table 5 and Figure 3 represent the variation of small scale parameter $\left(e_{0}a\right)$ on critical buckling load, whereas Figure 4 demonstrates the variation of small scale parameter $\left(e_{0}a\right)$ on the critical buckling load ratio. The critical buckling load ratio may be defined as the ratio of critical buckling load calculated using nonlocal theory and classical theory $\left(e_{0}a=0\right)$. This critical buckling load ratio acts as an index to estimate the influence of the small scale parameter $\left(e_{0}a\right)$ qualitatively on buckling load. From Table 5 and Figure 3, it is observed that critical buckling load is decreasing with an increase in small scale parameter $\left(e_{0}a\right)$, and this decline is more in case of the C-C boundary condition. From Figure 4, it may also be noted that the influence of the small scale parameter is comparatively more in C-C edge and less in C-F edge.

### 4.4. Influence of Aspect Ratio

**Figure 5:**
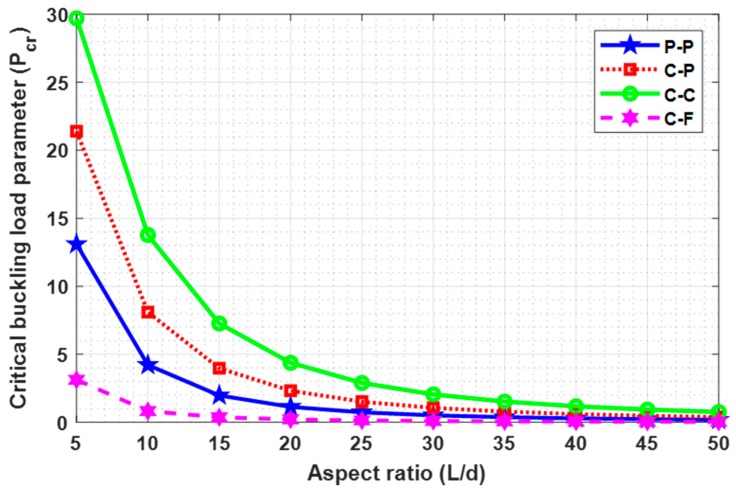
Variation of (L/d) with $\left(P_{cr}\right)$ for N=7, $e_{0}a=1$, and $H_{a}=2$.

The objective of this subsection is to study the impact of aspect ratio (L/d) on the critical buckling load $\left(P_{cr}\right)$ with “P-P, C-P, C-C, and C-F” boundary conditions for different L/d (5, 10, 15, 20, 25, 30, 35, 40, 45, 50). The effect of aspect ratio has been reported in Table 6 and Figure 5 for N=7, $e_{0}a=1$, and $H_{a}=2$, which are respectively. From this study, it is essential to note that the critical buckling load decreases with an increase in aspect ratio (L/d). This decrease is more consequential for the lower value of aspect ratio.

### 4.5. Influence of Hartmann Parameter

**Figure 6:**
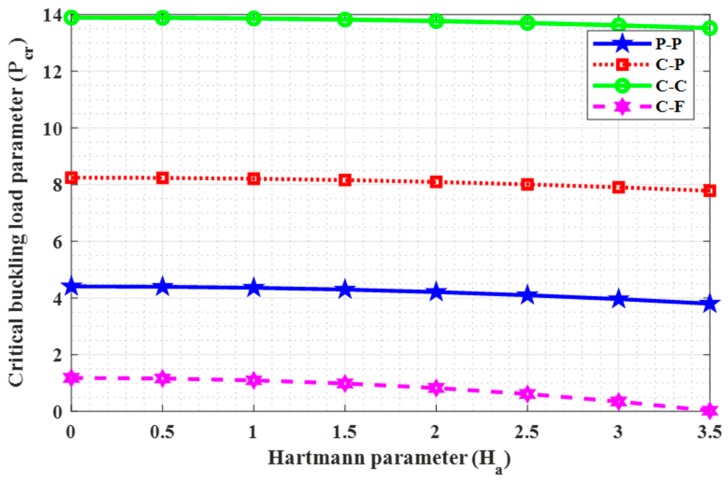
Response of $\left(H_{a}\right)$ on $\left(P_{cr}\right)$ for N=7, $e_{0}a=1$, and L=10.

For the designing of electromagnetic devices, proper knowledge about the effect of electric and magnetic fields on critical buckling load is necessary as it greatly influences the lifespan of electromagnetic devices. In this regard, the effect of Hartmann parameter $\left(H_{a}\right)$ on the critical buckling load $\left(P_{cr}\right)$ has been studied in this subsection for different values of $H_{a}$ (0, 1, 1.5, 2, 2.5, 3, 3.5). Table 7 and Figure 6 represent the results for the variation of critical buckling load with Hartmann parameter $\left(H_{a}\right)$ for “P-P, C-P, C-C, and C-F” boundary conditions. From these results, we may note that the critical buckling load decreases with increase in Hartmann parameter, but this drop in critical buckling load is very slow.

## 5. Buckling Mode Shape

**Figure 7:**
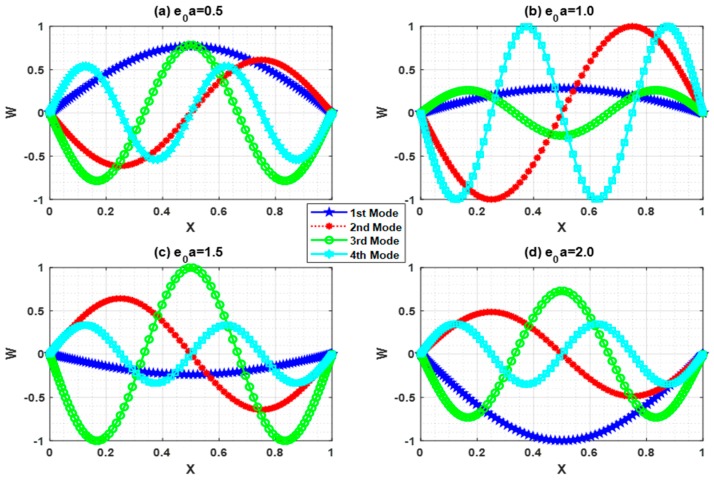
Buckling mode shape for P-P boundary condition with $H_{a}=0.5$ and L=10.

**Figure 8:**
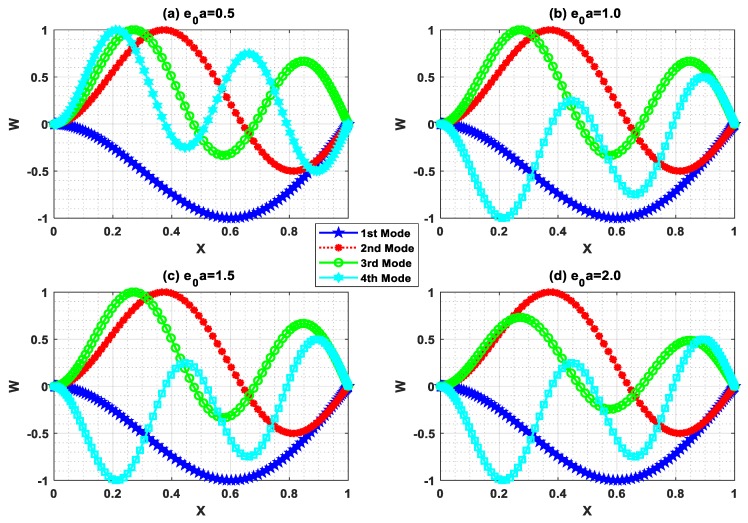
Buckling mode shape for C-P boundary condition with $H_{a}=0.5$ and L=10.

**Figure 9:**
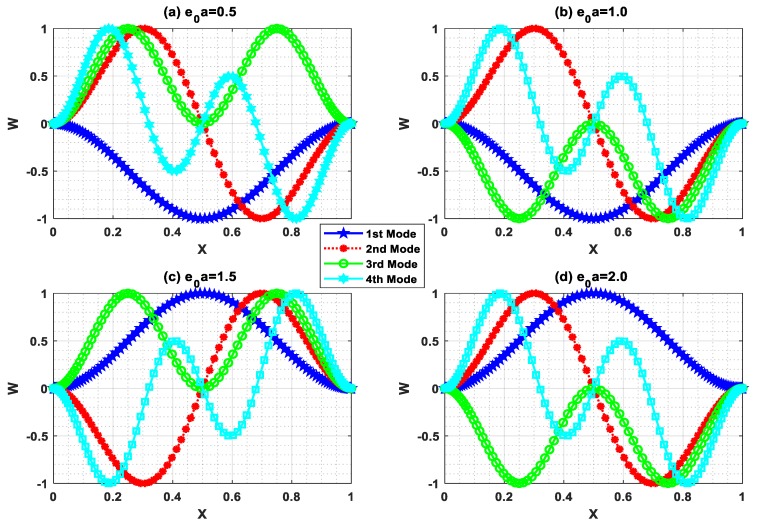
Buckling mode shape for C-C boundary condition with $H_{a}=0.5$ and L=10.

**Figure 10:**
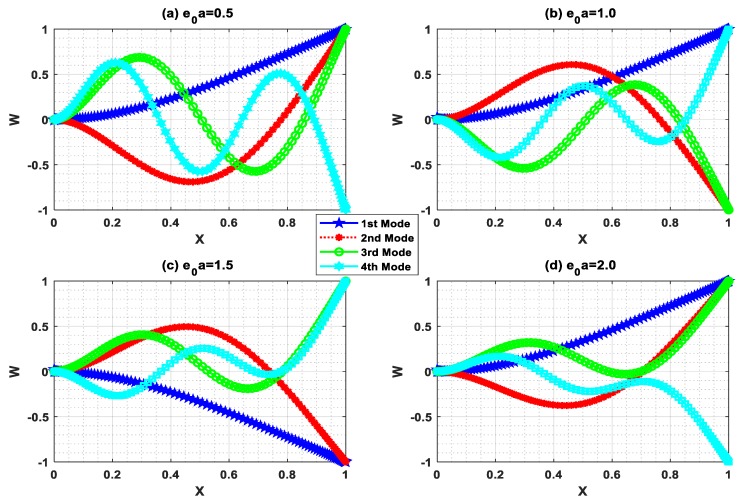
Buckling mode shape for C-F boundary condition with $H_{a}=0.5$ and L=10.

Buckling is the state of instability of structures that leads to structural failure. In this circumstance, the buckling mode shape has a vital role in predicting the instability. In this regard, buckling mode shapes were plotted with $H_{a}=0.5$ and L=10 for different $e_{0}a$ (0.5, 1, 1.5, 2). Figure 7, Figure 8, Figure 9 and Figure 10 show the buckling mode shapes for “P-P, C-P, C-C, and C-F” boundary conditions, respectively. From these figures, one may witness the sensitiveness of buckling mode shapes towards scaling parameters. Also, these mode shapes help to predict the mechanical health and lifespan of several electromechanical devices.

## 6. Concluding Remarks

The buckling behavior of Electromagnetic nanobeam is investigated in the combined framework of Euler–Bernoulli beam theory and Eringen’s nonlocal theory. Critical buckling load parameters were obtained using shifted Chebyshev polynomials-based Rayleigh-Ritz method for all the classical boundary conditions such as “Pined–Pined (P-P), Clamped–Pined (C-P), Clamped–Clamped (C-C), and Clamped-Free (C-F)”. The C-F boundary condition converges faster with N=5, whereas other boundary conditions such as P-P, C-P, and C-C require N=7 for achieving convergence to the desired accuracy. Critical buckling load parameters decrease with an increase in small scale parameter, and this decline is more in case of the C-C boundary condition. It may also be noted that the influence of small scale parameter is comparatively more in C-C edge and less in C-F edge. It is interesting to note that the critical buckling load decreases with an increase in aspect ratio. This decrease is more consequential for the lower values of aspect ratio. We may note that the critical buckling load decreases with an increase in Hartmann parameter, but this drop in critical buckling load is prolonged. The C-C nanobeam possesses the highest critical buckling load, whereas the C-F nanobeam possesses the lowest.

## Figures and Tables

**Table 1:** Values of p  and q for different boundary conditions [33,34].

**Boundary Conditions**	p	q
**P-P**	1	1
**C-P**	2	1
**C-C**	2	2
**C-F**	2	0

**Table 2:** Comparison of “Critical buckling load” $\left(P_{cr}\right)$ in nN with [15] for $\frac{L}{d}=10$.

**(**a**) Comparison of P-P and C-P boundary conditions**				
$e_{0}a$	**P-P**		**C-P**	
	**Present**	**[15]**	**Present**	**[15]**
0	4.8447	4.8447	9.9155	9.9155
0.5	4.7281	4.7281	9.4349	9.4349
1	4.4095	4.4095	8.2461	8.2461
1.5	3.9644	3.9644	6.8151	6.8151
2	3.4735	3.4735	5.4830	5.4830
**(**b**) Comparison of C-C and C-F boundary conditions**				
$e_{0}a$	**C-C**		**C-F**	
	**Present**	**[15]**	**Present**	**[15]**
0	19.3790	19.3790	1.2112	1.2112
0.5	17.6381	17.6381	1.2037	1.2037
1	13.8939	13.8939	1.1820	1.1820
1.5	10.2630	10.2630	1.1475	1.1475
2	7.5137	7.5137	1.1024	1.1024

**Table 3:** Comparison of “Critical buckling load” $\left(P_{cr}\right)$ in nN with Navier’s closed-form solution for P-P boundary condition.

$e_{0}a$	**Present (R-R)**	**Navier’s Solution**
0	4.7950	4.7950
0.5	4.6783	4.6783
1	4.3598	4.3598
1.5	3.9146	3.9146
2	3.4237	3.4237
2.5	2.9467	2.9467
3	2.5160	2.5160
3.5	2.1434	2.1434
4	1.8287	1.8287

**Table 4:** Effect of no. of terms (N) on critical buckling load $\left(P_{cr}\right)$ with $H_{a}=1$, L=10, and $e_{0}a=1$.

N	**P-P**	**C-P**	**C-C**	**C-F**
2	5.211151	8.452577	14.486540	1.100703
3	4.362030	8.240579	13.869377	1.094818
4	4.362029	8.209547	13.869177	1.094614
5	4.359792	8.208410	13.863597	1.094613
6	4.359790	8.208342	13.863594	1.094613
7	4.359791	8.208341	13.863584	1.094613
8	4.359791	8.208341	13.863584	1.094613
9	4.359791	8.208341	13.863584	1.094613
10	4.359791	8.208341	13.863584	1.094613

**Table 5:** Effect of small scale parameter $\left(e_{0}a\right)$ on critical buckling load $\left(P_{cr}\right)$ in nN with N=7, L=10, and $H_{a}=2$.

$e_{0}a$	**P-P**	**C-P**	**C-C**	**C-F**
0	4.645787	9.748924	19.229661	0.840199
0.5	4.529126	9.275797	17.497785	0.836210
1	4.210584	8.094859	13.772744	0.824504
1.5	3.765433	6.673148	10.160354	0.805815
2	3.274518	5.349719	7.425368	0.781223
2.5	2.797456	4.255716	5.510414	0.751973
3	2.366762	3.397759	4.184610	0.719306
3.5	1.994208	2.737457	3.253694	0.684313
4	1.679487	2.230108	2.585265	0.647847
4.5	1.416723	1.837632	2.093720	0.610487
5	1.198278	1.530786	1.723927	0.572534

**Table 6:** Effect of aspect ratio (L/d) on critical buckling load $\left(P_{cr}\right)$ in nN with N=7, $e_{0}a=1$, and $H_{a}=2$.

L/d	**P-P**	**C-P**	**C-C**	**C-F**
5	13.098075	21.398879	29.701473	3.124893
10	4.210584	8.094859	13.772744	0.824504
15	1.974312	3.972494	7.267483	0.370283
20	1.132281	2.318949	4.374446	0.209052
25	0.731275	1.510526	2.893474	0.134022
30	0.510359	1.059208	2.046611	0.093157
35	0.376087	0.782797	1.520631	0.068481
40	0.288505	0.601638	1.172838	0.052449
45	0.228261	0.476628	0.931406	0.041452
50	0.185069	0.386801	0.757197	0.033582

**Table 7:** Effect of Hartmann parameter $\left(H_{a}\right)$ on critical buckling load $\left(P_{cr}\right)$ in nN with N=7, $e_{0}a=1$, and L=10.

$H_{a}$	**P-P**	**C-P**	**C-C**	**C-F**
0	4.409527	8.246144	13.893850	1.182017
0.5	4.397093	8.236694	13.886284	1.160290
1	4.359791	8.208341	13.863584	1.094613
1.5	4.297621	8.161070	13.825741	0.983502
2	4.210584	8.094859	13.772744	0.824504
2.5	4.098678	8.009677	13.704575	0.614226
3	3.961904	7.905484	13.621213	0.348424
3.5	3.800262	7.782236	13.522631	0.022134
